# Personalized Nutrition Biomarkers and Dietary Strategies for Atherosclerosis Risk Management: A Systematic Review

**DOI:** 10.3390/nu17172804

**Published:** 2025-08-28

**Authors:** Khadijah Fayyaz, Muhammad Saeed ud Din, Husnain Bashir, Firdos Ahmad, Colin J. Barrow, Nauman Khalid

**Affiliations:** 1 Department of Human Nutrition and Dietetics, School of Food and Agricultural Sciences, University of Management and Technology, Lahore 54000, Pakistan; khadijah.fayyaz@umt.edu.pk (K.F.); saeed.uddin@umt.edu.pk (M.S.u.D.); 2 Department of Cardiology, Sheikh Zayed Medical Complex, Lahore 54560, Pakistan; husnain.bashir@skzmdc.edu.pk; 3 Department of Basic Medical Sciences, College of Medicine, University of Sharjah, Sharjah 27272, United Arab Emirates; fahmad@sharjah.ac.ae; 4 Centre for Sustainable Bioproducts, Deakin University, Waurn Ponds, VIC 3217, Australia; 5 College of Health Sciences, Abu Dhabi University, Abu Dhabi 59911, United Arab Emirates

**Keywords:** personalized nutrition, atherosclerosis, nutrigenomics, microbiome, metabolome, biomarkers

## Abstract

**Background/Objectives**: Atherosclerosis is a major contributor to ischemic cardiovascular diseases (CVDs) such as myocardial infarction and stroke, which are leading causes of mortality and morbidity. The management of atherosclerosis through personalized nutrition has gained importance in recent years due to advancements in nutrigenomics, gut microbiome evaluation, and metabolomics. However, no systematic review has comprehensively evaluated the impact of personalized nutrition interventions on atherosclerotic plaque progression and clinical outcomes in humans. **Methods**: We adopted a systematic approach based on Preferred Reporting Items for Systematic Reviews and Meta-Analyses (PRISMA) guidelines. Key databases like PubMed, Cochrane, Google Scholar, and MEDLINE via EBSCOhost were searched using predefined terms related to personalized nutrition, atherosclerosis, nutrigenomics, and clinical outcomes. **Results**: Evidence evaluation using the framework of Boffetta et al. for cumulative evidence on the joint effects of genes and environments strongly suggested significant diet–gene interactions. Polymorphisms in the apolipoprotein A-II (*APOA2*) gene have been shown to influence body mass index and lipid levels. Furthermore, studies have demonstrated that omega-3 polyunsaturated fatty acids (PUFAs) can modulate microRNA expression, thereby impacting lipid metabolism. Epigenetic studies showed that dietary components can modify histone acetylation and non-coding RNA activity, which ultimately influence gene expression related to inflammation and lipid metabolism, improving clinical outcomes in atherosclerosis management. **Conclusions**: Integrating personalized nutrition into clinical practice promises to enhance atherosclerosis outcomes through targeted dietary interventions. Advancements in personalized nutrition offer a promising pathway toward more effective and personalized approaches to cardiovascular health.

## 1. Introduction

Cardiovascular diseases (CVDs) pose a significant threat to public health worldwide and are the leading cause of mortality. Atherosclerosis leads to the development of various CVDs, such as coronary heart disease, stroke, and myocardial infarction. Every year, approximately 17.9 million individuals die due to CVDs, accounting for 32% of all fatalities [1]. Of these, up to 80% of deaths occur in low- and middle-income countries [2].

Atherosclerosis is a chronic inflammatory condition involving impaired lipid metabolism, arterial stiffness, foam cell formation, and blood vessel obstruction [3]. The disease is no longer restricted to Western countries but is now a significant contributor to mortality worldwide. Recently, the incidence of atherosclerosis has drastically increased in young individuals, particularly females, belonging to diverse ethnicities [4]. In addition, the risk factors that were previously known to increase the chances of atherosclerosis have also changed over time. For example, recent studies have questioned the cardio-protective effects of high-density lipoproteins (HDL). There is also new evidence that triglyceride-rich lipoproteins and low-density lipoproteins are causal hallmarks for the development of atherosclerosis [5].

The pathogenesis of atherosclerosis has been the subject of extensive research. In addition to traditional lifestyle factors like diet, exercise, smoking, alcohol consumption, climate, and geography, non-traditional drivers of atherosclerosis, such as environmental exposure, genetic characteristics, and individual gut microbiomes, are also now recognised as key contributors to disease risk [6].

The development of atherosclerosis is significantly influenced by genetics. The factors contributing to this include genetic variations such as gene mutations and polymorphisms associated with metabolic disorders, as well as epigenetics. This has been demonstrated in studies involving twins and other populations [7]. In particular, for monogenic familial hypercholesterolemia (FH) cases, atherosclerosis can be inherited following a Mendelian inheritance pattern. This is because the mutations in the low-density lipoprotein receptor (LDLR) in FH can lead to an increased level of cholesterol in the plasma. This, in turn, increases the susceptibility to developing atherosclerosis in FH patients [8]. Researchers have discovered that the gut microbiome also plays a role in the development of atherosclerosis. The microorganisms in the gut can influence the physiological system by regulating the immune and endocrine systems, among others. Metabolites derived from these bacteria, including short-chain fatty acids (SCFAs), choline, and trimethylamine (TMA), influence the metabolism of bile acids, cholesterol, and trimethylamine N-oxide (TMAO), thereby impacting inflammatory pathways within the individual [9]. Thus, understanding the causal factors leading to atherosclerosis can significantly improve its prevention and treatment. This was emphasized in a study by the World Health Organization (WHO), which stated that 75% of CVDs in their early stages can be prevented by managing the associated risk factors, thereby reducing the health burden on individuals and the healthcare system [10].

The most important factor in reducing the risk of atherosclerosis is diet [11]. Various international guidelines, in particular, the National Institute for Health and Care Excellence (NICE) guidelines, the European Society of Cardiology (ESC) guidelines, and guidelines from the American Heart Association (AHA) and the American College of Cardiologists (ACC) have provided the dietary recommendations to manage CVDs. The dietary recommendations provided by these organizations overlap, to a large extent. However, a few disparities exist owing to a lack of consensus and limited evidence [12]. These guidelines promote low-fat diets and Mediterranean eating patterns. In particular, dietary recommendations emphasize the reduction of trans and saturated fats, sodium, and processed foods, while encouraging the consumption of fruits, vegetables, whole grains, and lean proteins. The AHA recommends regular aerobic exercise, while the ESC provides moderate and vigorous exercise guidelines [12].

Personalized nutrition has emerged as a promising nutritional intervention approach for combating a range of diseases, including atherosclerosis. While there is currently no universally agreed-upon definition, in this review, we adopt the definition proposed by the American Nutrition Association, which describes personalized nutrition as “a field that leverages human individuality to drive nutrition strategies that prevent, manage, and treat disease and optimise health” [13]. This definition considers multiple factors, including an individual’s genetics, gut microbiome, and other unique characteristics [14]. Research has evidenced that nutrient metabolism is influenced by genetics, thereby increasing or decreasing an individual’s susceptibility to disease. Additionally, telomere length, circadian rhythm, and chrono-nutrition contribute substantially to the development of metabolic diseases [15]. Thus, effective implementation of personalized nutrition is contingent upon several factors, including genetic analysis, an in-depth evaluation of the gut microbiome, and individual dietary behaviours.

There have been recent important advances in understanding the pathogenesis, underlying risk factors, and dietary prevention strategies for atherosclerosis. However, despite these advances, the level of agreement on existing guidelines and strategies to effectively tailor and implement personalized nutrition remains elusive. This review aims to systematically search, identify, and provide a narrative synthesis of the studies that evaluate the effectiveness of personalized nutrition interventions, guided by genetic, microbiome, and metabolome profiling, compared to standard dietary guidelines, for preventing and managing atherosclerosis in adult populations. By synthesizing the current evidence, this review seeks to elucidate the potential of personalized dietary strategies in mitigating atherosclerosis risk, considering the complex interplay of individual genetic predispositions, gut microbiota composition, metabolomic markers, and dietary habits.

## 2. Methodology

### 2.1. Study Design

This study adopted a systematic approach to review the literature. This systematic review followed the Preferred Reporting Items for Systematic Reviews and Meta-Analyses (PRISMA) methodology (Table S1). The PRISMA framework consists of a 27-item checklist and a four-phase flow diagram.

### 2.2. Eligibility Criteria for Study Inclusion

Inclusion and exclusion criteria were defined as per the PICO framework (Table 1). This review was limited to studies of adults aged 18 years and older diagnosed with atherosclerosis or otherwise identified as being at high risk. The studies eligible for this review included primary studies dealing with either observational (case–control, prospective, retrospective, cross-sectional), experimental (randomized, non-randomized controlled trials), or qualitative study designs. In vitro studies involving gene–diet interactions were considered as supportive evidence. To ensure that the review was based on the latest findings, the timeline for study inclusion was set at ten years, which also captures advancements in biomarker technology, dietary assessment methods, and personalized nutrition approaches. Exclusion criteria included studies with the paediatric population (<18 years); studies in which dietary effects could not be isolated due to concurrent drug therapy, and studies not focusing on genotype, biomarker, or omics outcomes. In this review, we adopt the definition of personalized nutrition proposed by the American Nutrition Association, as “a field that leverages human individuality to drive nutrition strategies that prevent, manage, and treat disease and optimise health” [13]. This definition reflects the broad, integrative nature of the field, encompassing genetic, phenotypic, microbiome, behavioural, and environmental factors, while grounding it in clinical relevance. Studies that did not comply with the inclusion criteria were not included in this review.

### 2.3. Search Strategy and Identification of Eligible Studies

Databases including PubMed, Cochrane, Google Scholar, and MEDLINE via EBSCOhost were used to gather articles for this systematic review. The following keywords and their combinations (using Boolean operands) were used to collect the most relevant literature related to atherosclerosis and personalized nutrition:

((“Personalized Nutrition” [All Fields] OR “Precision Nutrition” [All Fields]) AND (“Atherosclerosis” [MeSH Terms] OR “Atherosclerosis” [All Fields] OR “Cardiovascular Disease” [All Fields]) AND (“Dietary Strategies” [All Fields] OR “Nutritional Intervention” [All Fields] OR “Evidence-Based Nutrition” [All Fields]) AND (“Lipids” [All Fields] OR “Inflammation” [All Fields] OR “Oxidative Stress” [All Fields]) AND (“Omics” [All Fields] OR “Genetic” [All Fields] OR “Epigenetic” [All Fields] OR “Microbiome” [All Fields] OR “Metabolites” [All Fields])). After retrieving studies that met the inclusion criteria, a detailed screening process followed. After the initial screening of studies, the reviewers independently checked the full texts to determine which studies were eligible.

### 2.4. Data Extraction Process

The compilation of studies was performed according to established inclusion and exclusion criteria. Pertinent data from each study included study design, country, sample population, personalized nutritional interventions, and measurable outcomes. Measurable outcomes included clinical and biochemical markers such as lipid profiles (LDL-C, HDL-C, TG, TRLs), inflammatory markers (hs-CRP, homocysteine), and metabolic indicators (bile acids, acetoacetate, carnitines). Molecular outcomes included telomere length, miRNA and mRNA expression, and DNA methylation (*LINE-1, Alu*). Genotype-specific responses to diet were assessed through SNPs (e.g., *APOE, CD36, CLOCK, PLA2, MGLL, CDKN2A/B*) and genotype risk scores. Dietary intake and adherence to interventions were also included when linked to relevant health outcomes.

Following the PRISMA guidelines and the methodological recommendations of Zeng et al. [16], two independent reviewers screened the titles and abstracts of all identified studies. The same two reviewers independently reviewed full-text articles of potentially eligible studies to determine final inclusion. In cases where there were discrepancies or disagreements during any stage of study selection, a third reviewer was consulted. Consensus by discussion was used to resolve any disagreements among the reviewers.

### 2.5. Data Synthesis

Data were synthesized in a tabular format, summarizing key intervention characteristics, outcomes, and biomarkers from the included studies (Table 2). For each study, relevant clinical and biochemical markers, molecular outcomes, and genotype-specific responses were recorded. In cases where sufficient data were available, a meta-analysis was performed to combine the results of multiple studies and assess overall trends. The meta-analysis used a random-effects model, and statistical heterogeneity was assessed. Forest plots were created to visually display pooled results and confidence intervals. For studies where meta-analysis was not feasible, results were synthesized narratively, comparing the direction and magnitude of observed effects across different interventions.

### 2.6. Quality Appraisal and Risk of Bias Assessment of Studies

The Critical Appraisal Skills Programme (CASP) tool was used to evaluate the quality of the included studies. The CASP tool is a criterion-based assessment instrument for evaluating the methodological rigor and quality of the studies [31]. The reviewers used questions in the tool to analyse the appropriateness of the study protocols, as well as the presentation and significance of the results. Moreover, studies were assessed for risk of bias using the Cochrane Risk of Bias tool for randomized studies (RoB 2.0) and RoB in non-randomised studies (ROBINS-E and ROBINS-I) (Figures S1–S3). These tools contain a pre-set collection of bias domains that allow the researchers to focus on different aspects of research design in randomized and non-randomized studies [32]. Quality appraisal using these tools found that most randomized controlled trials were of moderate-to-high quality, with low risk of bias across essential domains, while observational and cross-sectional studies were generally of moderate quality because of residual confounding and limited causal inference. For example, the Mediterranean diet–telomere study [22] and CORDIOPREV [18] displayed high scores among large RCTs. Cross-sectional and cohort studies were rated as only moderate because of their correlational design limitations. Intervention studies regarding omega-3 and PUFA supplementation were consistently rated at moderate quality, indicating considerable associations with biomarkers (Figures S1–S3).

Moreover, each study was evaluated using the framework of Boffetta et al. [33] evaluating the cumulative evidence on the joint effects of genes and environments for evidence in nutrigenomics, which considers study design, reproducibility, biological plausibility, strength, and consistency of associations (Table S2). This approach allowed for the transparent classification of the evidence into strong, moderate, or weak categories.

## 3. Results

### 3.1. Study Selection

**Figure 1:**
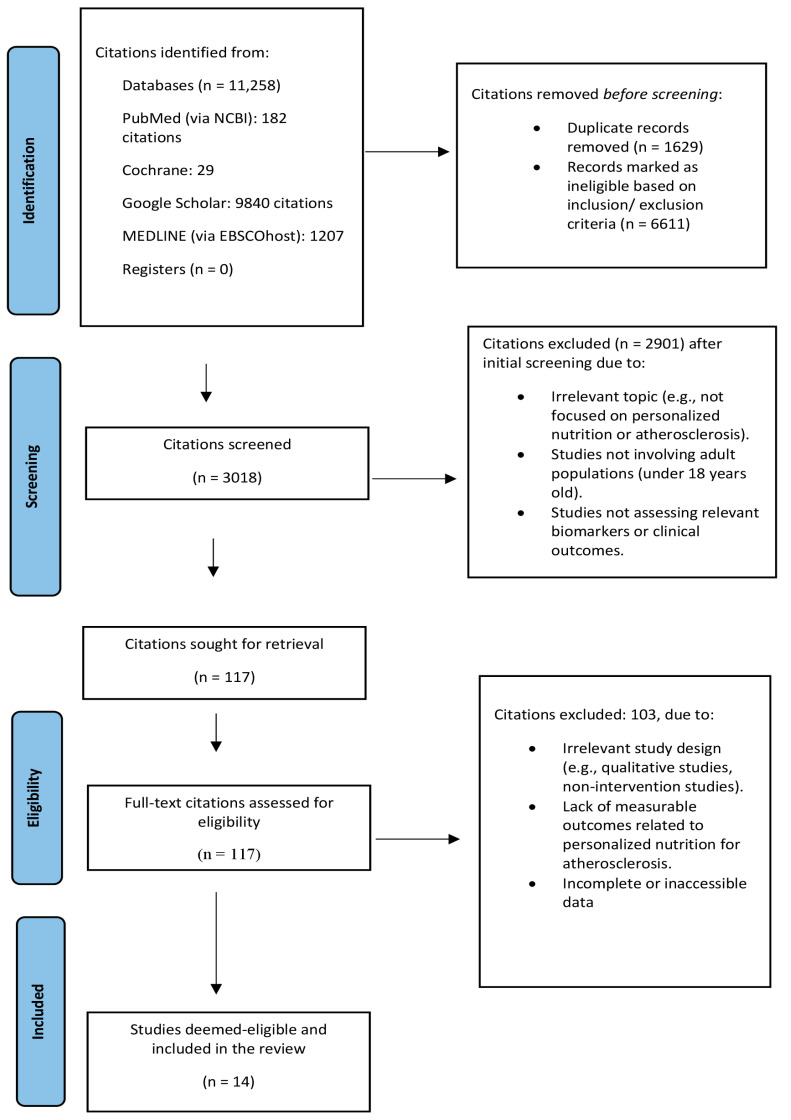
PRISMA flow diagram depicting the identification, screening, and selection of studies for this systematic review.

A total of 11,258 citations were found in the database search. After removal of 1629 duplicates, a total of 9629 records remained. Of these, 6611 were marked as ineligible based on the defined inclusion and exclusion criteria. The remaining 3018 records were screened by title and abstract, from which 2901 were excluded based on irrelevance to the review topic. A total of 117 full-text articles were assessed for eligibility. Of these, 103 were excluded due to lack of relevant dietary intervention comparisons, inappropriate populations (e.g., population < 18 years), or insufficient outcome data. In total, 14 studies met the inclusion criteria and were included in this review. The PRISMA flow diagram (Figure 1) illustrates how the studies for this systematic review were identified, screened, and selected.

### 3.2. Characteristics of the Included Studies

Table 2 summarizes the characteristics of the 14 high-quality studies included in this systematic review. The studies were conducted across multiple countries, including Spain (two studies), Canada (multiple studies from Quebec), China, the United States, and Iran. The included studies employed a variety of research designs, such as randomized controlled trials, cross-sectional analyses, genotype–diet interaction trials, and mechanistic interventional studies. The populations studied were equally diverse, encompassing middle-aged adults at cardiovascular risk, older adults, and population-based cohorts. Most studies comprised mixed-sex participant groups, with sample sizes ranging from 99 to 12,065 individuals. Data collection techniques varied, including clinical laboratory testing, dietary interventions, genotype profiling, biomarker assessments (e.g., triglycerides, hs-CRP, lipoproteins), and self-reported dietary intake questionnaires. Several studies also incorporated postprandial testing protocols and omics-based modelling approaches (e.g., transcriptomics, methylation, miRNA expression), strongly emphasizing personalized risk stratification.

### 3.3. Summary of Findings

A consistent theme across the studies was the inter-individual variability in TG response to omega-3 PUFA supplementation, which was modulated by genetic variants. Notably, research conducted in Quebec (*n* = 208) identified significant associations between polymorphisms in genes such as *PLA2G2A, PLA2G2C, PLA2G4A, MGLL, IQCJ, NXPH1, PHF17*, and *MYB* and changes in TG or LDL-C levels post supplementation [27]. Genotype risk scores explained that genetic variations, and *IQCJ*, *NXPH1*, *PHF17*, and *MYB* genes accounted for nearly 50% of the variation in TG response, emphasizing the potential for gene-guided personalized nutrition strategies [27].

**Figure 2:**
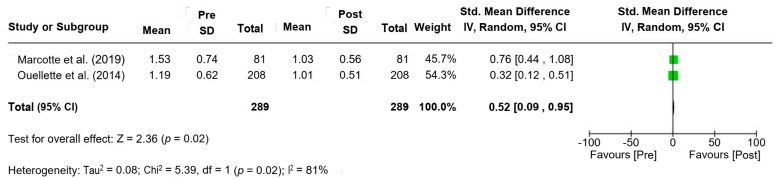
Forest plot illustrating the standardized mean difference (SMD) in triglyceride (TG) levels before and after omega-3 PUFA supplementation [19,28].

Sub-group analysis (Figure 2) examining the effect of omega-3 PUFA supplementation on TG levels demonstrated a statistically significant reduction in TG following supplementation (SMD = 0.52; 95% CI: 0.09 to 0.95; *p* = 0.02), although substantial heterogeneity was observed (I^2^ = 81%). These findings suggest that while omega-3 supplementation effectively reduces TG levels, the degree of response is significantly influenced by individual genetic makeup [19,28].

The CORDIOPREV trial from Spain highlighted the effects of *APOE* [18] and *CLOCK* gene [24] variants on lipid and inflammatory outcomes in coronary heart disease (CHD) patients following Mediterranean and low-fat diets (<30% of total calories). *APOE rs439401* T-allele carriers displayed a significantly greater reduction in postprandial TG and large TRLs compared to the results for CC genotype individuals (*p* < 0.03), while *CLOCK rs4580704* C/C genotype subjects showed superior reductions in hs-CRP and improved HDL/*ApoA1* ratios [24].

**Figure 3:**
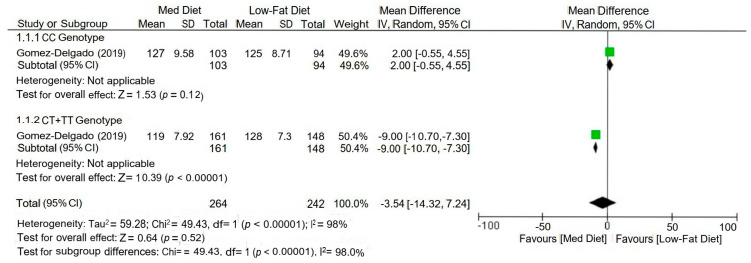
Forest plot comparing the effect of Mediterranean diet (MedDiet) versus low-fat diet on lipid outcomes across different genotypes [18].

Sub-group meta-analysis based on genotype demonstrated that the effectiveness of the Mediterranean diet compared to a low-fat diet varied significantly. The Mediterranean diet did not result in a statistically significant improvement in individuals with the CC genotype, with a mean difference of 2.00 [95% CI: −0.55 to 4.55] (*p* = 0.12). However, among those with the CT or TT genotypes, the Mediterranean diet led to a significantly greater reduction in the outcome measure, with a mean difference of −9.00 [95% CI: −10.70 to −7.30] (*p* < 0.00001). When both subgroups were combined (Figure 3), the overall effect was not statistically significant (mean difference = −3.54 [−14.32 to 7.24], *p* = 0.52), and substantial heterogeneity was observed (I^2^ = 98%). However, the test results for subgroup differences were highly significant (Chi^2^ = 49.43, df = 1, *p* < 0.00001), suggesting a strong gene–diet interaction.

The high heterogeneity observed in our pooled analyses (*I*^2^ = 81–98%) highlights the complexity of diet–gene interactions. Differences in genetic background can partly explain this variability, along with dietary intervention type (e.g., fish oil capsules versus dietary PUFA intake), baseline lipid levels, and population characteristics such as ethnicity and health status.

### 3.4. Personalized Biomarkers in Atherosclerosis

Beyond traditional biomarkers for atherosclerosis diagnosis, recent research has identified some personalized biomarkers based on genetics, microbiomes, and metabolomes [34]. A study in China found that compared to traditional models for subclinical atherosclerosis (SA) based on postprandial biomarkers, an omics-based model (ROC AUC = 91%) shows a higher potential to indicate SA and subsequent management of atherosclerosis [17]. These biomarkers (Table 3) provide more sophisticated evidence for the early diagnosis of atherosclerosis.

#### 3.4.1. Genetic Biomarkers

Genetic differences are significant (approximately 40%) in atherosclerosis [6]. The Human Genome Project showed that all persons share 99.9% commonality in their genetic sequences; the remaining sequences differ by polymorphic regions, including single-nucleotide polymorphisms (SNPs), accounting for most of these differences. These variations are particularly interesting in genome-wide association studies (GWAS), which help determine genotype–phenotype correlations. Genes responsible for the development of complex and prevalent forms of coronary artery disease (CAD) have been identified through GWAS. Furthermore, more than 200 loci for CAD have been identified through extensive cohort studies in Japan and Europe, including CARDIOGRAM and UK BIOBANK [41]. These identified loci constitute only a small portion of the disease’s heritable component, implying that many genes remain unidentified. While some variations have been found to have significant effect sizes, most loci show weak effects that cannot be useful in isolation for predicting the risk. Furthermore, each gene makes only a very small contribution to the overall susceptibility. However, the cumulative effect of these genes explains most of the genetically inherited factors [6].

##### Lipoprotein (a)

High cholesterol concentration in the blood is caused by excessive low-density lipoprotein (LDL), the primary risk factor for atherosclerosis. These levels are influenced by numerous genetic factors, including 1500 different GWAS signals and significant environmental factors [42]. In terms of this, lipoprotein (a) has become an important factor in developing atherosclerosis. Recent data from genetic and epidemiological studies suggest that lipoprotein (a) may be an inherited risk factor predisposing individuals to atherosclerosis-related CVDs [43,44,45]. Guidelines from Europe and the US suggest that using an Lp(a) threshold of 50 mg/dL is a risk multiplicator necessary to improve understanding of each person’s forecasted ten-year risk for CVD due to atherosclerosis. This recommendation is based on the 2010 EAS statement [43]. Recent studies suggest that having an Lp(a) level of 100mg/dL (approximately 250 nmol/L) increases the risk of atherosclerosis twofold [46].

Although Lp(a) levels are primarily determined by genetics, researchers have indicated that dietary modifications can modestly and inconsistently affect their levels. A consistent observation is the opposite response of Lp(a) and LDL cholesterol (LDL-C) to diets low in dietary saturated fatty acids (SFA). Controlled feeding trials and meta-analyses show that lowering SFA reduces LDL-C but modestly increases Lp(a) concentrations [47].

A recent meta-analysis based on 27 randomized controlled trials with 49 dietary comparisons was conducted to quantify the association of SFA intake with Lp(a) levels. On average, a 5.5% increase in Lp(a) levels was observed in relation to low SFA diets compared to high SFA diets [35]. However, these studies used carbohydrate- or trans fatty acid (TFA)-based diets as an SFA replacement. Replacing SFAs with monounsaturated or polyunsaturated fatty acids did not yield consistent results. Similarly, a GET-READI trial conducted on the African-American population showed that consuming a DASH-style low-SFA diet resulted in a 24% increase and 10% decrease in Lp(a) and LDL-C levels, respectively [47]. These clinically paradoxical responses are of critical importance in populations with inherently elevated Lp(a) levels, as following population-wide recommendations for reducing SFA will have an opposite effect on Lp(a) levels [47].

Research has also revealed the positive impact of the ketogenic diet on Lp(a) concentration. In a crossover trial, a decrease in Lp(a) concentrations from 401 U/L at baseline to 286 U/L was noted after two weeks of following a ketogenic diet [48]. These findings suggest that diet does have an impact on Lp(a). However, the findings remain inconsistent. Further studies involving metabolomics and lipidomic analyses are required to evaluate how diet impacts Lp(a) levels.

##### MicroRNA and Non-Coding RNA

Non-coding RNAs (lncRNAs) and microRNAs (miRNAs) play a considerable role in controlling atherogenic plaque development. MicroRNAs destroy messenger RNA as the main post-transcriptional regulatory mechanism, which is employed to control gene expression levels. Thus, the expression levels of miRNAs define the pathophysiological basis for atherogenesis. For instance, high levels of miR-29, miR-100, miR-155, miR-199, miR-221, miR-363, miR-497, miR-508, mi-21, miR-34, miR-210, miR-146, and miR-181 have been detected in atherosclerotic coronary arteries, while miR-1273, miR-490, miR-24, and miR-1284 are scarce and present in low quantities [49].

A long-term high-fat diet (HFD) causes the upregulation of miR-142-3p, miR-142-5p, miR-21, miR-146a, and miR-146b and suppresses others such as miR-200b, miR-200c, and miR-122, reflecting broad alterations in adipose tissue metabolism [50].

The onset of atherosclerosis begins with endothelial dysfunction, which prompts an inflammatory process leading to foam cell formation. Furthermore, miR-10a has been shown to block *GATA6/VCAM1* signalling in endothelial cells (ECs), thereby protecting against atheroma. Teixeira et al. (2022) stated that miR-10a, miR-31, and miR-17-3p regulate inflammation and the expression of adhesion molecules in ECs, while miR-126, one of the most researched miRNAs, prevents atherosclerosis by reducing endothelial permeability and modifying the vascular endothelial growth factor (VEGF) pathway [51]. In cellular experiments, arachidonic acid (AA) increased the expression of anti-inflammatory microRNAs such as miR-10a, miR-17-3p, miR-125a, miR-155, and miR-181b in endothelial cells under basal conditions [23]. Also, endothelial activation is suppressed by miR-146a, which induces endothelial nitric oxide synthase (eNOS) synthesis, leading to reduced lipid uptake by macrophages, indicating its anti-atherogenic properties [52]. Docosahexaenoic acid (DHA) has been found to suppress miR-146a under inflammatory stimulation [23]. Research has shown that DHA supports an anti-inflammatory endothelial state, while AA exhibits more context-dependent effects, upregulating anti-inflammatory miRNAs under basal conditions but generally acting as a precursor to pro-inflammatory eicosanoids [53]. Variants in genes encoding the fatty acid desaturase enzymes FADS1 and FADS2 affect an individual’s ability to convert given precursors into AA, EPA, and DHA. For example, it has been suggested that the *FADS1 rs174547* polymorphism diminishes desaturase activity and therefore, the endogenous production of EPA and DHA. Due to the variation in the FADS genotype and dietary balance of ω-6 to ω-3 among individuals, personalized dietary recommendations can benefit such individuals [53].

Furthermore, improper synthesis and non-coding RNA activity affect atherosclerosis development [54]. There is a growing recognition that long non-coding RNA (lncRNA) contributes significantly to the atherosclerotic environment. In addition, it can activate or repress genes on a transcriptional and translational basis. Some long non-coding RNAs (LncRNAs) play a role in reducing senescence and DNA damage of atheroma plaques in pigs and humans [54].

**Figure 4:**
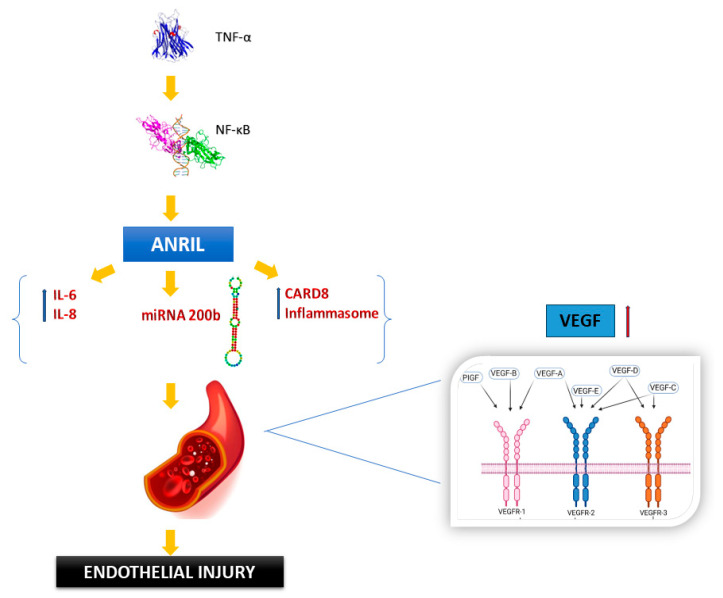
Mechanism depicting the role of ANRIL in atherosclerosis. Distinct transcripts of the ANRIL gene protect and promote atherosclerosis development and progression.

Another significant molecule involved in the development of atherosclerosis is Antisense Non-Coding RNA in the *INK4 Locus* (*ANRIL*). The more severe the atherosclerosis, the higher the level of this molecule in the body. It is expressed in various cell types within the atherosclerotic plaque [37]. Circular ANRIL (*circANRIL*) has just been identified (Figure 4) as different from its linear counterparts. Levels of *circANRIL* are inversely related to atherogenic status, and CircANRIL is located in smooth muscle cells, macrophages, and vascular tissue as an anti-atherogenic agent [37]. A study was conducted to assess the impact of a high-fat diet on LncRNAs in an atherosclerosis *ApoE*(−/−) mouse model [55]. It was found that 354 LncRNAs were affected by HFD, with 186 downregulated and 168 upregulated. These changes were consequently associated with inflammatory pathways in lipid metabolism and other vascular alterations, highlighting the role of diet in modulating LncRNA landscape [55].

#### 3.4.2. Microbiome-Associated Biomarkers

Health professionals and scientists have investigated how the microbiome impacts the development of atherosclerosis. The gut microbiome in people with atherosclerosis is different from that of healthy individuals, particularly with regard to the levels of *Enterobacteriaceae* and *Streptococcus* species, which tend to be higher in individuals with atherosclerosis. The gut microbiota produces vital molecules important for transport and metabolic functions that ensure a healthy cardiovascular system, and so differences in the microbiome can impact cardiovascular health, with some factors promoting atherosclerosis [56]. Gut bacteria metabolize food components, which means that different diets impact the gut metabolome. For example, Western dietary patterns rich in red meat, eggs, and dairy supply choline and L-carnitine, which produce trimethylamine (TMA) as the intestinal microbiota breaks down choline. On the other hand, diets rich in plant sterol esters and β-glucan have been found to reduce TMA levels, consequently preventing atherosclerosis [57]. TMA can convert to trimethylamine-N-oxide (TMAO), an atherogenic substance produced by the liver. Atherosclerosis is facilitated by increased arterial inflammation and reactive platelet activity that can be promoted by TMAO [57].

In addition to TMAO, other pro-atherosclerotic compounds are also produced by Gram-negative bacteria and are represented by the molecular class known as lipopolysaccharides (LPS). LPS can promote numerous inflammatory reactions involved in atherosclerosis. Activation of nicotinamide adenine dinucleotide phosphate (NADPH) oxidase by LPS leads to the release of reactive oxygen species (ROS). The stimulation by the gut microbiota produces secondary bile acids, which in turn trigger two important receptors: membrane-bound Takeda G protein-coupled receptor 5 (TGR5) and nuclear Farnesoid X receptor (FXR) [57]. These receptors have also been reported to reduce the rate of atherosclerosis progression via NF-κB suppression that causes attenuation of pro-inflammatory cytokines production and inhibits cluster of differentiation (CD36) expression, thus reducing LDL absorption [57]. However, the gut microbiota produces short-chain fatty acids (SCFAs) like butyrate from the fermentation of dietary fibre, which has been shown to suppress the formation of atherosclerotic plaques. The potential of butyrate to improve plaque stability is derived from its ability to reduce ROS and nitric oxide release by macrophages and suppress the synthesis of well-established inflammatory molecules like matrix metalloproteinase-2, chemotaxis protein-1, and endothelial cell adhesion molecule-1. Therefore, consumption of a high-fibre diet is linked to increased levels of SCFAs (e.g., butyrate) [57].

In a recent study investigating the relationship between the gut microbiome of patients with atherosclerotic CVD and clinical characteristics, a random forest classifier was implemented for 405 atherosclerotic and control samples. The classifier demonstrated encouraging diagnostic potential with respect to an Area Under the Receiver Operating Curve (AUC) score that stands at 0.86 [56]. The classifier discovered significant bacteria, including *Lachnospiraceae*, *Clostridium nexile*, *R. gnavus*, *A. parvulum*, *E. coli*, *E. lenta*, and *L. salivarius*. These findings suggest that gut microbiome characteristics connected to atherosclerosis can be used as non-invasive biomarkers.

Further probing showed that the classifier founded on microbial lipid genes (MLGs) performed better than the one founded exclusively on trimethylamine (TMA) lyases (AUC 0.63), indicating that atherosclerosis is due to multiple factors, not just TMAO [56,58]. Researchers found certain types of bacteria in high abundance in individuals with atherosclerosis, which encode for the enzyme choline-TMA lyase, among them *S. anginosus*, *C. nexile*, and unclassified *Erysipelotrichaceae. E. aerogenes* and *Klebsiella pneumoniae*, which are bacterial species with different genes encoding choline-TMA lyase and another more promiscuous types of TMA lyase.

Some bacteria are observed to exhibit virulence components, including immunogenic lipoprotein A (IlpA) and PhoP within the PhoQ/PhoP system, which can play a role in the development of atherosclerosis [56]. It is therefore important to understand the connection between the gut microbiome and distinct metabolic reactions to enable a transition to a more personalized mode of healthcare.

#### 3.4.3. Metabolomic Biomarkers

The metabolomic approach assesses small molecule metabolites in biological samples that reflect the state of the system or the entire organism and offers additional insights into the pathology of the illness [59].

The application of metabolomic techniques has successfully unveiled evidence of risk factors associated with atherosclerosis. Research conducted on human tissues has identified metabolites and pathways that could be linked to atherosclerosis [60]. These metabolites provide insight into metabolic deregulation associated with augmented plaque formation in aortic tissue; for instance, sphingolipids, ceramides, and glycerophospholipids are some of the metabolites discussed in the literature. Atherosclerosis was characterized by variation in purine pathway-related metabolites, including phosphatidylethanolamine-ceramides (PE-Cer) and glucosylceramides (GlcCers) [61].

Two pathways known to participate in the development of atherosclerosis, namely ceramide and cholesterol, may share different lipids with previously unknown links to atherogenesis, specifically phosphatidylethanolamine-ceramides (PE-Cers) [61]. Lipidomic analysis of atherosclerotic plaques has revealed significant accumulation of PE-Cers, indicating their importance in sphingolipid and sterol metabolism. Functionally, PE-Cer performs a dual role, i.e., it affects the membrane structure by altering lateral cholesterol organization and modulates the pro-inflammatory signalling cascade. This dual influence on both membrane architecture and intracellular signalling positions PE-Cer as a mechanistic bridge between ceramide-driven stress responses and cholesterol-driven lipid accumulation [61,62].

Glycosphingolipids (glucosylceramide and lactosylceramide), which are derived from ceramides, are reported to be present in high levels in human atherosclerotic plaques and are pro-inflammatory [63]. Lactosylceramide and ceramide induce cell apoptosis through neutral sphingomyelinase. Lactosylceramide has also been suggested to promote plaque formation. It upregulates the adhesion molecules on vSMCs and vascular endothelial cells and activates neutrophils, thereby facilitating plaque inflammation [63].

**Figure 5:**
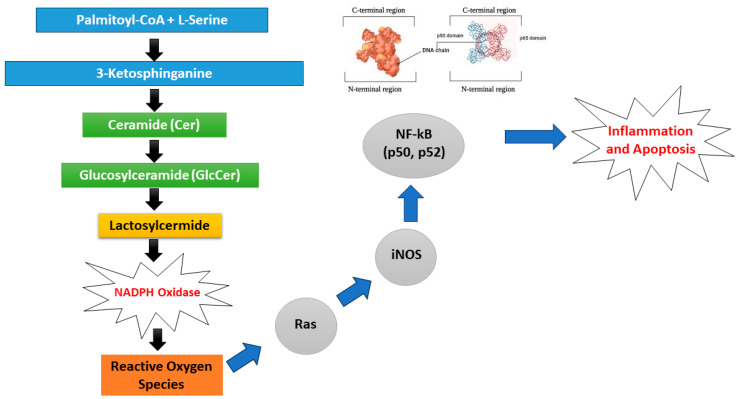
LacCer activates NADPH oxidase, creating ROS and with heightened oxidative stress, generating a significant number of reactive oxygen species that trigger various kinds of signalling molecules, followed by cascade receptivity; as a result, many different phenotypes begin to emerge (including atherosclerosis).

The conversion of GlcCers, which are glycosphingolipids, from Cer to lactosylceramides (Figure 5) is part of a mechanism that causes cell division, which is triggered by oxidized low-density lipoproteins [61]. Unusual buildup of glycosphingolipids, particularly GlcCers, has been found in atheromas in the human aorta. In the course of atherogenesis, increased oxidative low-density lipoproteins (LDL) may, in turn, raise GlcCers, leading to inflammation, thus worsening the condition [64].

Diet plays a significant role in modulating sphingolipid metabolism and atherosclerosis risk. Research using experimental models has shown that trans fats from the diet are incorporated into sphingolipids via the serine palmitoyltransferase pathway, leading to the formation of ceramides and glycosphingolipids [65]. Dietary trans-fat intake promotes the production and secretion of sphingolipids and very-low-density lipoproteins (VLDL), accelerating plaque formation compared to the results for diets rich in unsaturated fats [65].

### 3.5. Navigating Personalized Nutrition Approaches: Tailoring Strategies for Atherosclerosis Management

Over the years, international associations have provided scientifically proven nutrition advice to manage risk factors for atherosclerosis and CVDs. Despite these efforts, residual risk remains due to variability across studies and statistical approaches [66]. Notable differences exist between the AHA and ESC guidelines: the AHA provides detailed recommendations regarding specific foods and quantities, while the ESC focuses on nutrient-based targets [67]. Ideally, clinical guidelines should be grounded in multiple RCTs, but evidence remains limited. An assessment revealed that only 10% of ACC/AHA and 15% of ESC guidelines are supported by high-quality RCTs [68]. Similarly, the NICE relies on older RCTs for fibre intake recommendations, while the ESC incorporates meta-analyses up to the 2010s [69]. For saturated fat, the ESC relies on modelling data, whereas the AHA does not specifically mention atherosclerosis [12].

This limits the ability of health practitioners to provide precise clinical recommendations to patients due to a lack of consensus regarding available data and comprehensive nutrition recommendations. At present, the prevention of atherosclerosis requires dietary guidance developed through assessing epidemiologic and interventional research to arrive at a consensual guideline based on the quality of the evidence [67].

At an individual level, population-based nutrition guidance might not always work efficiently. Genetic predispositions, metabolic phenotype, health condition, lifestyle choices, and life events of each patient will determine whether some food elements should be enhanced, reduced, or eliminated to optimize one’s risk profile [70,71,72]. Atherosclerosis is a disease whose course is driven by the inflammation-related genes, the production of foam cells, the accumulation of cholesterol by macrophages, and cholesterol metabolism [73]. Food components directly or indirectly influence various forms of genome behaviour (genes and proteins) [21,74]. Thus, each person’s genetic constitution can determine the overall health effects of consuming a particular diet.

It has been observed that some foods exhibit epigenetic changes via actions of the short non-coding RNA molecules, histone acetylation/deacetylation processes, and DNA methylation (methionine, folic acid, B vitamins) [75]. It is reported that increased levels of homocysteine in the blood are related to higher DNA methylation levels, offering additional evidence of genetic influence on the methylation patterns that may play a role in atherosclerosis progression. The long interspersed nuclear element-1 (LINE-1) methylation was 0.05 (0.01, 0.13) 5 mC higher for every 3 mmol/L increase in homocysteine [20]. Previous research findings in mouse models indicate a relationship between hypomethylated DNA and reduced methylenetetrahydrofolate reductase (MTHFR) and DNA methyltransferase (DNMT) gene expression during atherosclerosis development phases [76,77]. These other two genes, whose activity partially depends on DNA methylation, are associated with the initial development of atherosclerosis.

Moreover, studies have also shown that individuals with the CC genotype of apolipoprotein A2 (*APOA2*) rs5082 polymorphism in the gene promoter region exhibit a significantly larger body mass index (BMI) than those with the TT genotype, consuming diets rich in saturated fats [78]. Similarly, recent research indicates that the same gene–nutrient interaction may lead to higher levels of low-density lipoprotein cholesterol (LDL-C) and increased LDL-C/HDL-C ratio among diseased subjects. Consequently, a low-SFA diet will benefit people with the CC genotype for the *APOA2* SNP, thus preventing them from becoming obese and developing hypercholesteremia [78,79].

Even though these studies focused on individual macronutrients, dietary pattern comparisons are becoming increasingly common to account for the synergistic effect of meals. Two groups were created for the 12-month Coronary Diet Intervention with Olive Oil and Cardiovascular Prevention (CORDIOPREV) intervention trial. One group adhered to a Mediterranean diet with 35% fat content, consisting of 22% monounsaturated fatty acid, while another group was placed on a ‘low-fat’ diet containing 8% fat and 12% monounsaturated fatty acid [18,80]. The research showed that in the study sample, the TG levels of those with the T allele of the APOE gene were lower than the levels in those with the TG genotype. A higher level of HDL-C was observed in people with the T allele than in those without it. Research findings have shown why individuals respond differently to similar diets by indicating interactions between genetics and food [80]. Another study was conducted to determine whether there were effects of the Mediterranean diet on telomere lengths in persons at high cardiovascular risk, determining that the pro12Ala variant in the PPARγ2 gene (rs1801282) was associated with better maintenance of telomere lengths for people following the Mediterranean diet [22]. In the CORDIOPREV study, CLOCK gene variants were associated with the Mediterranean diet in patients with coronary heart disease (CHD). Individuals harbouring polymorphic rs4580704 CLOCK genes displayed better inflammation markers and lipid profiles when they adhered to the Mediterranean diet [23].

Previous cases using SNP examinations showed that there are diet–gene interactions. Some of this research explored the interaction of omega-3 fatty acids and fish oil supplementation with SNP in *PLA2*, *MGLL*, *IQCJ*, *NXPH1*, *PHF17*, and *MYB* genes and found that variations in these genes influence TG levels in plasma post-supplementation [27]. Nevertheless, the pathophysiology of risk factors such as obesity and dyslipidaemia is most likely polygenic and intricate [81]. Individuals are awarded scores using the Genetic Risk Score (GRS) method, depending on the count of risk alleles and the trait under consideration. Those in the high-risk group (with a score greater than 7) displayed a 1.94 cm larger waist circumference and BMIs averaging 0.93 kg/m^2^, approximately 1.69% higher relative to the results for low-risk individuals (with a score = 7), in addition to higher body fat index values [79].

Additionally, there were observed anomalies within certain dietary areas when comparing both clusters based on their rates, including a greater number of cases involving acute myocardial infarction among those at higher risks, as opposed to those with lower chances who consumed polyunsaturated fatty acids (PUFAs). These prediction algorithms may serve as the basis of stratification, allowing high-risk individuals to alter their food intake based on their genotype. For instance, a 31-SNP GRS was developed using imputation from the 1000 Genomes Project database for predicting non-responsiveness to omega-3 PUFA treatment aimed at reducing TG levels [19,79,82]. Also, genetic risk scores (GRS) accounted for almost 50% of the difference in TG response to omega-3 supplementation, highlighting the importance of genetic predisposition in personalized omega-3 interventions in atherosclerosis management [21]. Research has also shown that by substituting SFAs with PUFAs, peripheral blood mononuclear cells showed a downregulation of uncoupling protein 2 (*UCP2*) and peroxisome proliferator-activated receptor delta (*PPARD*) genes, which are implicated in inflammation [25].

Chromatin is a nucleosome made up of histones and the DNA fragments that wrap around them. Changing the DNA strand’s ability to be transcribed requires the addition of acetylation, methylation, phosphorylation, and ubiquitination chemicals at the histone tail [83]. Histone acetylation, for instance, can unravel the chromatin and open the DNA for transcription, generally leading to gene expression. However, bioactive curcumin protects the heart by blocking the acetylation of particular histones, turning off genes that promote vascular inflammation, like *TREM-1*, in CVDs [84]. The reason for mRNA degradation and inhibition of mRNAs translation into proteins is the binding of microRNAs (miRNAs) to messenger RNAs, referred to as non-coding RNAs. An example is when omega-3 PUFAs regulate microRNA quantity that control lipid metabolism genes [50]. It has been shown that omega-3 (*n*-3-PUFA) and arachidonic acid (*n*-6-PUFA) supplementation affects miRNA expression in endothelial and monocyte/macrophage cell lines, thus influencing vascular inflammation [23].

### 3.6. Personalized Nutrition in Clinical Settings: Opportunities and Challenges

Healthcare professionals can combine population-based advice with personalized nutrition strategies to devise food-oriented plans to maximize benefits and minimize risks for each individual. This can include providing specific dietary advice based on knowledge of different molecular mechanisms affected by other variables like microbiota or epigenetic factors, in addition to new genetic variations, and closely watching any responses following food intake [85]. Healthcare professionals can use personalized nutrition strategies to identify patients with high blood pressure who possess certain gut bacterial species or epigenetic markers that indicate they are sensitive to dietary salt [86]. Consequently, the appropriate dietary plans will be uniquely proposed for each patient, improving the treatment’s outcome and efficiency. Therefore, health practitioners can turn general dietary advice into more tailored therapies through personalized nutrition approaches.

Epigenetic studies involving diet treatment are uncommon and usually focus on DNA methylation, with histone modifications and miRNAs in cellular or animal models being the most studied pathways [87]. More research is needed to show how certain diets can lead to gene changes predisposing individuals to atherosclerosis. However, this is challenging due to the fact that some of these markers change over time. Furthermore, as epigenetic marks are often tissue-specific, it is necessary to employ oral or blood cells as markers of epigenetic processes in tissues that are difficult to reach. Diverse sequencing approaches are essential when dealing with various epigenetic markers (i.e., DNA, RNA, proteins), making this process relatively complicated and costly [87]. As a result, the current drawbacks of epigenomics limit its immediate potential to contribute to a precise nutrition strategy for atherosclerosis prevention.

The practical application of nutrigenomic methods in clinical and biological research is associated with several problems. The AHA scientific statement sheds light on several challenges and limitations, particularly in setting up and conducting randomized clinical trials that can offer a firm basis of information for patient consultations [88]. Among the main disadvantages of nutrigenomic clinical trials is that controlling subjects’ meals becomes extremely challenging. This issue might be partially solved by identifying specific biomarkers related to food consumption, allowing us to determine whether eating habits are related to abnormalities. Furthermore, actual problems with regulation occur, especially when fixing terminology in clinical practice and nutrigenomics/nutrigenetics studies. For instance, in the postgenomic era, the gene (as the unit of heredity) has been modified so that now it means more than just a DNA sequence but also includes the way chromosomes are organized and the way that other non-DNA macromolecules contribute to epigenetic regulation [89]. Many ethical, legal, and societal obstacles exist in regards to protecting nutrigenomic information and ensuring that marginalized countries have equitable access to nutritional resources [90].

Given the current challenges and limitations, the most effective strategies for integrating nutrigenomics into healthcare systems remain uncertain. However, one potential application could involve physicians recommending personalized diets for patients with non-communicable diseases. A recent literature review has supported the efficiency of the Mediterranean diet in protecting against heart disease. Moreover, while the Mediterranean diet is seen as part of the solution to the current high rate of non-communicable diseases, it is not a replacement for creating personalized nutrition programs [91].

Achieving personalized nutrition requires integrating nutrition into nutrigenomics and nutrigenetics into medicine. This requires scientific research and clinical studies, including randomized clinical trials, to create a strong foundation of knowledge that will enable clinicians to counsel patients effectively [85]. Customized nutrition professional development is also important, as is creating counselling therapeutic principles that inform evidence-based practices [85].

A potential limitation of this review is the restriction to studies published within the last ten years. While this approach ensures the inclusion of the most recent evidence and advancements in personalized nutrition and omics-based interventions for atherosclerosis, it may have excluded relevant earlier foundational studies. Moreover, our review highlighted inter-individual variability in gene–diet interactions. However, subgroup analyses by sex and age were not consistently available across the included studies. As a result, our review did not stratify outcomes by these demographic factors.

## 4. Conclusions and Future Directions

Intervention guidelines provided by the AHA, ESC, and NICE are globally recognized for treating atherosclerosis and emphasize dietary habits and physical activity. These guidelines generally agree on the need to decrease the consumption of saturated fats, sugars, and sodium, while increasing the consumption of fruits, vegetables, whole grains, and good fats. Experimental data, including clinical studies, strongly support these guidelines, but they need to be updated and revised periodically due to the diversity of recommendations and the dynamic nature of dietary research.

The findings of this review suggest that following Mediterranean and low-fat diets consistently improves lipid profiles, reduces systemic inflammation, and supports vascular health. Specific nutrient strategies, such as increasing omega-3 polyunsaturated fatty acids and lowering saturated fat intake, further help lower cardiovascular risk. Importantly, the effectiveness of these dietary interventions is influenced by inter-individual variability, including genetic predispositions, gut microbiome composition, and metabolomic signatures. Factors like genetic background, gut microbiome composition, and metabolic profiles play a role. Therefore, while general dietary guidelines offer a strong foundation, using personalized nutrition approaches allows us to tailor prevention strategies more effectively, maximizing their impact on reducing atherosclerosis risk.

Even though personalized nutrition is still in its infancy, some outcomes have been implemented into mainstream medicine, particularly in the nutrigenetics sector. The substantial amount of data supporting a genetic-based strategy necessitates further advances in this area. The application of nutrigenomics in practice is hampered by some specific factors, including dietitians’ attitudes towards genetic tests and the absence of personalized nutrition education in medical programs aimed at health specialists. The personalized nutrition model relies upon synergistic collaboration among different players within the system, making adoption slow.

Nutrition experts need to adopt innovative diagnosis and monitoring techniques, while policymakers should develop appropriate strategies to protect personal data obtained through extensive data analysis. Private industries also possess significant responsibilities to ensure that personalized nutrition can be effectively translated and incorporated into public nutrition practices. In its position paper, the International Society of Nutrigenetics and Nutrigenomics emphasizes the critical need to address ethical and legal considerations when introducing personalized nutrition into society. These considerations should include issues related to equity, environmental factors, the interplay between genetics and food choices, individual gene–lifestyle interactions, and the influence of the microbiome. This is particularly important for CVDs, and atherosclerosis in particular, where there is clear evidence that dietary interventions are critical for both its prevention and treatment.

## Figures and Tables

**Table 1:** Inclusion and exclusion criteria used for drafting the systematic review.

Criteria	Inclusion	Exclusion
Population	Adults (≥18 years) with diagnosed atherosclerosis or at high risk of CVD.	Children, adolescents (<18 years), or unrelated populations (e.g., cancer patients, non-CVD conditions).
Intervention	Personalized nutrition interventions considering individual omics profiling (genetics, microbiome, metabolomics, epigenetics).	Generalized or non-personalized dietary advice.
Comparison	Standard care diets or control groups.	Studies without a comparison group.
Outcomes	Measured effects on atherosclerosis-related clinical outcomes, biomarkers, or metabolic profiles.	Studies that do not measure or report relevant clinical, metabolic, or biomarker outcomes.
Study Design	Primary studies only: randomized controlled trials (RCTs); cohort studies; case–control, cross-sectional studies with analytical focus.	Narrative reviews, systematic reviews, meta-analyses, opinion pieces, and conference abstracts without full papers.
Language	Studies published in English.	Non-English publications without available translations.
Publication Date	Studies published within the last 10 years (to capture contemporary personalized nutrition advancements).	Studies older than 10 years.

**Table 2:** Summary and characteristics of the included studies.

**Study**	**Country**	**Sample/Population**	**Study Design**	Sample Size	Intervention	Duration	Outcome Measured	Gene/Genetic Variant Tested	Main Findings
[17]	China	Healthy subjects with and without subclinical atherosclerosis (SA)	Cross-sectional exploratory study	100 Chinese subjects (46 female, 54 male)	Mixed-meal test (low-fat (energy% < 30%) frozen meal)	2 weeks	164 blood biomarkers	Omics model	SA could not be accurately predicted using models, and it depends only on fasting biomarkers or baseline clinical characteristics. Conversely, an omics model based on the timing and quantity of postprandial biomarkers showed excellent performance [ROC AUC: 91%; 95% CI: 77–100].
[18]	Spain	CHD Patients	CORDIOPREV study (RCT)	506 (male = 433, female = 73)	Mediterranean diet and Low-fat diet	3 years	Postprandial TG and TRLs	*APOE rs439401, rs440446, rs7412*	Using a gene–diet approach, the study analysed the interaction between the *APOE rs439401* SNP and the MedDiet. Compared to CC patients, those in the MedDiet group who were carriers of the T-allele displayed a more significant decrease in postprandial triglycerides (TG: *p* = 0.03), as well as large triacylglycerol-rich lipoproteins (TRLs) TG (large TRLs TG; *p* = 0.01. Both the TG area under the curve (AUC-TG; P-interaction = 0.03) and the AUC-large TRLs TG (P-interaction = 0.02) showed consistent patterns that were significantly lower in T-allele carriers compared with levels in CC subjects.
[19]	Quebec, Canada	Caucasian subjects	Interventional	208	Omega-3 supplementation	2 years, 3 months	Plasma lipids	16 SNPs in *IQCJ*, 34 in *NXPH1*, 8 in *PHF17*, and 9 in *MYB*	The genotype risk score (GRS) accounted for 49.73 percent of the variation in TG response (*p* < 0.0001) in a general linear model that adjusted for age, sex, and body mass index.
[20]	New York and Los Angeles, USA	White, Caucasian Black, African-American Hispanic	Cross-sectional	987 (male: 469, female: 518)	-	2 years	Plasma homocysteine, micronutrients	Long interspersed nucleotide 1 (*LINE-1*) and *Alu*	The LINE-1 methylation was 0.05 (0.01, 0.13), %5 mC higher for every 3 mmol/L increase in homocysteine. Furthermore, a positive correlation (*p* trend = 0.03) was found between BMI and *LINE-1* methylation. The *LINE-1* was 0.35 (0.03, 0.67), %5 mC higher in participants with a 40 kg/m^2^ BMI than in those with a normal BMI. A variation of 0.10 (0.02, 0.19), %5 mC in *Alu* methylation was also noted for every 10 cm of height.
[21]	Quebec, Canada	Middle-aged adults at higher risk of developing chronic diseases	Cross-sectional	CARTaGENE biobank (12,065)	**-**	6 years	Lipid profile and intakes (%kcal/day) of total, saturated (SFA), monounsaturated (MUFA), and polyunsaturated (PUFA) fatty acids	*CD36* gene	Habitual fat consumption is linked to CD36 variations, which might explain later relationships with biomarkers associated with chronic diseases. Higher consumption of SFA was linked to *rs1054516* and *rs3173798* (both *p* < 0.05), and *rs1054516* was also linked to higher levels of serum triglycerides (*p* = 0.0065).
[22]	Spain	Patients without a history of cardiovascular disease but at high cardiovascular risk	Mediterranean diet for primary prevention of cardiovascular diseases (Prevención con DietaMediterránea) randomized trial	521	Mediterranean Diet	5 years	Telomere length	*PPARγ2* locus, *rs1801282*, *Ala* allele	After five years of follow-up, the *pro12Ala* polymorphism is linked to TL homeostasis in persons at high cardiovascular risk. Furthermore, among Ala carriers, a stronger defence against telomere shortening is provided by a higher level of adherence to the MeDiet pattern.
[23]	Germany	Monocyte/macrophage cell line RAW264.7 and the endothelial cell line TIME	In vitro experiment	-	Docosahexaenoic acid (DHA; *n*-3-PUFA) or arachidonic acid (AA; *n*-6-PUFA)	-	miRNAs	miRNAs	PUFAs affect miRNA expression in both cell types under investigation, regardless of the presence of an inflammatory stimulant. Moreover, it was shown that cellular PUFA enrichment had an impact on certain miRNAs previously connected to vascular inflammation.
[24]	Spain	CHD Patients	CORDIOPREV study (RCT)	897	Low-fat (LF) diet and Mediterranean diet (MedDiet)	12 months	hs-CRP, HDL/ApoA1	*CLOCK* SNPs *(rs1801260*, *rs3749474*, *rs4580704)*	The LF diet and the *rs4580704* SNP interact to improve the inflammation and dyslipidaemia associated with CHD. Compared to minor G allele carriers (G/G + C/G), major allele carriers C/C showed a higher drop in high-sensitivity C-reactive protein (*p* < 0.001) and a substantial rise in HDL/apolipoprotein A1 ratio (*p* = 0.029).
[25]	Oslo, Norway	Adults	RCT	99	PUFAs	8 weeks	Lipoprotein subclasses, bile acids, proprotein convertase subtilisin/kexin type 9, acetate, and acetoacetate	mRNA levels of *LXRA* and *LDLR*, *UCP2*, and *PPARD*	Subclasses of lipoproteins, myristoyl- and palmitoyl-carnitine, and kynurenine decreased when PUFAs were substituted for SFAs. On the other hand, the intervention raised the levels of acetoacetate, bile acids, proprotein convertase subtilisin/kexin type 9, and acetate. The intervention also changed a few amino acids. After substituting SFAs with PUFAs, peripheral blood mononuclear cells showed a drop in the mRNA levels of *UCP2* and *PPARD* and an increase in the mRNA levels of *LXRA* and *LDLR*, along with many genes implicated in inflammation and liver X receptor alpha target genes.
[26]	Quebec, Canada	Adults from Quebec Family Study (QFS)—observational	Quebec Family Study (QFS)—observational	541	-	-	Total fat intake; LDL-PPD	SNPs from a genome-wide association study (GWAS)	There is an interaction between dietary fat consumption and various SNPs in terms of variation in the LDL-PPD.
[27]	Quebec, Canada	Adults (18–50 years)	Interventional study	208	3 g/day of *n*-3 PUFA	6 weeks	TG levels	5 SNPs in *PLA2G2A*, 6 in *PLA2G2C*, 8 in *PLA2G2D*, 6 in *PLA2G2F*, 22 in *PLA2G4A*, 5 in *PLA2G6*, and 9 in *PLA2G7* were genotyped	These results suggest that SNPs in *PLA2* genes could influence plasma TG levels when supplemented with *n*-3 PUFA.
[28]	Quebec, Canada	Adults (18–50 years)	Interventional study	210	5 g/d of a fish oil supplement	6 weeks	LDL-C, particle size	18 SNPs of the *MGLL* gene	After supplementation with *n*-3 PUFA, plasma LDL-C levels and particle size may be modified by polymorphisms in the *MGLL* gene.
[29]	Quebec, Canada	Adults (18–50 years)	Interventional study	208	5 g/day of fish oil	6-week	Plasma TG	SNPs: *IQCJ, NXPH1, PHF17* and *MYB* genes	Using fine-mapping at GWAS-associated loci, SNPs partially explaining the significant interindividual heterogeneity in plasma TG levels induced by an *n*-3 FA supplementation were identified.
[30]	Iran	Adults	Mashhad Stroke and Heart Atherosclerotic Disorders (MASHAD) cohort study	1165	-	7 years	CVD risk, lipids	*CDKN2A/B-rs10811661* locus	A strong correlation was observed between cardiovascular risk variables and dyslipidaemia, as well as the *CDKN2A-rs10811661* polymorphism.

Abbreviations: AA = Arachidonic Acid; *ApoA1* = Apolipoprotein A1; APOE = Apolipoprotein E; CHD = Coronary Heart Disease; CI = Confidence Interval; *CLOCK* = Circadian Locomotor Output Cycles Kaput; CRP = C-Reactive Protein; CVD = Cardiovascular Disease; DHA = Docosahexaenoic Acid; GWAS = Genome-Wide Association Study; GRS = Genetic Risk Score; HDL-C = High-Density Lipoprotein Cholesterol; LDL-C = Low-Density Lipoprotein Cholesterol; LDL-PPD = Low-Density Lipoprotein Peak Particle Diameter; *LINE-1* = Long Interspersed Nuclear Elements-1; LF = Low-Fat; MedDiet (MeDiet) = Mediterranean Diet; *MGLL* = Monoglyceride Lipase; miRNA = MicroRNA; MUFA = Monounsaturated Fatty Acids; *n*-3 PUFA = Omega-3 Polyunsaturated Fatty Acids; *n*-6 PUFA = Omega-6 Polyunsaturated Fatty Acids; PUFA = Polyunsaturated Fatty Acids; *PPARγ2* = Peroxisome Proliferator-Activated Receptor Gamma 2; QFS = Quebec Family Study; RCT = Randomized Controlled Trial; SA = Subclinical Atherosclerosis; SFA = Saturated Fatty Acids; SNP = Single Nucleotide Polymorphism; TG = Triglycerides; TRLs = Triacylglycerol-Rich Lipoproteins.

**Table 3:** Evidence-based, personalized biomarkers associated with atherosclerosis.

Biomarker Categories	Biological Markers	Mechanism/Aspects of Personalized Nutrition	Outcome	References
Genetic Markers	Lipoprotein (a)	The LPA gene, in particular, plays a major role in determining Lp(a) levels, but other treatments have also been proven to affect them.	Lp(a) significantly increases the risk of ASCVD that remains after statin treatment in individuals.	[34]
	Mutations in PCSK9 (proprotein convertase subtilisin/kexin type 9) Protein	Lowers the amounts of LDLR expressed in peripheral tissues or the liver, which indirectly obstructs the absorption of LDL by hepatocytes and other tissues.	Interference with molecular pathways during the onset and development of atherosclerotic plaque	[35,36]
	Antisense noncoding RNA in the INK4 locus (ANRIL)	Regulate the division and death of cells	Change the arterial plaque size and the apoptotic debris removal process	[36]
	CDKN2A/2B Rs10811661 (C/T) polymorphism	A TT genotype has been linked to a higher risk of CVD, insulin resistance, and hypercholesterolemia. These effects were more noticeable in the subgroup with low physical activity levels and high dietary energy intake.	Genetic variation increases the risk of cardiovascular disease and dyslipidemia.	[30]
	ANRIL	Modify chromatin to control the growth of vascular smooth muscle cells (VSMCs) in plaques. Additionally, alter transcriptional levels to impact macrophage proliferation and death.	Atherosclerotic plaque growth is tightly linked to the proliferation and death of related cells.	[37]
	Circulating miRNAs	A PUFA-enriched normocaloric diet is linked to modifications in the circulating profile of miRNA. A high-fat diet demonstrated how TGRL uses miRNA to sway the endothelium pro-inflammatory response.	Decreased miR-21, miR-30, miR-126 and miR-221-3p; and increased miR-21, miR-92a and miR-99a with the progression and degradation of atherosclerosis phenotypes	[38,39,40]
			*miR-24* *miR-122* *miR-185* *miR-223* *miR-486* Cholesterol homeostasis and reverse cholesterol transport	
			*miR-155* *miR-378a* Plaque rupture in atherosclerosis	

## Data Availability

The data is available upon request from the authors.

## Supplementary Materials

The following supporting information can be downloaded at: https://www.mdpi.com/article/10.3390/nu17172804/s1. Table S1: PRISMA Checklist for drafting a systematic review on Personalized Nutrition Biomarkers and Dietary Strategies for Atherosclerosis Risk Management. Table S2: Evaluation of Gene–Diet Interaction Studies Using the Boffetta et al. Framework. Figure S1: Traffic light plot for non-randomized observational studies [17,20,21,26]. Figure S2: Traffic light plot for randomized controlled trial [18,22,24,25]. Figure S3: Traffic light plot for non-randomized interventional studies [19,27,28,29].
